# Mu-desynchronization, N400 and corticospinal excitability during observation of natural and anatomically unnatural finger movements

**DOI:** 10.3389/fnhum.2022.973229

**Published:** 2022-09-02

**Authors:** Nikolay Syrov, Dimitri Bredikhin, Lev Yakovlev, Andrei Miroshnikov, Alexander Kaplan

**Affiliations:** ^1^Baltic Center for Artificial Intelligence and Neurotechnology, Immanuel Kant Baltic Federal University, Kaliningrad, Russia; ^2^V. Zelman Center for Neurobiology and Brain Restoration, Skolkovo Institute of Science and Technology, Moscow, Russia; ^3^Department of Human and Animal Physiology, Faculty of Biology, M. V. Lomonosov Moscow State University, Moscow, Russia; ^4^Department of Psychology, Centre for Cognition and Decision Making, National Research University Higher School of Economics, Moscow, Russia

**Keywords:** action observation, event-related desynchronization, N400, corticospinal excitability, mirror-neuron system, mu-rhythm, unnatural human movements

## Abstract

The action observation networks (AON) (or the mirror neuron system) are the neural underpinnings of visuomotor integration and play an important role in motor control. Besides, one of the main functions of the human mirror neuron system is recognition of observed actions and the prediction of its outcome through the comparison with the internal mental motor representation. Previous studies focused on the human mirror neurons (MNs) activation during object-oriented movements observation, therefore intransitive movements observation effects on MNs activity remains relatively little-studied. Moreover, the dependence of MNs activation on the biomechanical characteristics of observed movement and their biological plausibility remained highly underexplored. In this study we proposed that naturalness of observed intransitive movement can modulate the MNs activity. Event-related desynchronization (ERD) of sensorimotor electroencephalography (EEG) rhythms, N400 event-related potentials (ERPs) component and corticospinal excitability were investigated in twenty healthy volunteers during observation of simple non-transitive finger flexion that might be either biomechanically natural or unnatural when finger wriggled out toward the dorsal side of palm. We showed that both natural and unnatural movements caused mu/beta-desynchronization, which gradually increased during the flexion phase and returned to baseline while observation of extension. Desynchronization of the mu-rhythm was significantly higher during observation of the natural movements. At the same time, beta-rhythm was not found to be sensitive to the action naturalness. Also, observation of unnatural movements caused an increased amplitude of the N400 component registered in the centro-parietal regions. We suggest that the sensitivity of N400 to intransitive action observation with no explicit semantic context might imply the broader role of N400 sources within AON. Surprisingly, no changes in corticospinal excitability were found. This lack of excitability modulation by action observation could be related with dependence of the M1 activity on the observed movement phase.

## Introduction

Neural networks providing the ability to plan and control own movements, as well as to interpret the movements of other people, are directly related to the visual-sensorimotor integration (Bernstein, 1947; Schack, 2004). They are likely to condition the therapeutic potential of such rehabilitation practices as motion observation therapy and mirror therapy (Altschuler et al., 1999; Ertelt et al., 2007; Rizzolatti et al., 2009; Zhu et al., 2015). Importantly, action observation networks (AON) or mirror neuron systems are the neural substrate of such visual-sensorimotor integration processes (Condy et al., 2021). These networks demonstrated activation both during movement execution and during observation of another person’s action (Rizzolatti et al., 1996; Fabbri-Destro and Rizzolatti, 2008; Condy et al., 2021).

The execution of movement is always accompanied by perceptual feedback: Either visual, tactile, or kinesthetic. A constant connection between a movement and its perceptual features leads to the formation of a mental representation of motor action, a multisensory model of movement stored in long-term memory (Schack, 2004; Schack and Mechsner, 2006). Thus, the “mirroring” ability of these networks is the result of correlated sensorimotor learning (Gillmeister et al., 2008; Heyes, 2011; Cooper et al., 2013; Cook et al., 2014).

It has been considered that mirror neurons (MNs) are sensitive to purposeful actions and play the role of the predictors of one’s action. The results of invasive neural activity registrations with monkeys are in line with this hypothesis (Rizzolatti et al., 2001; Fabbri-Destro and Rizzolatti, 2008). At the same time, other studies (Hickok, 2013; Condy et al., 2021) claim that functioning of mirror systems in humans might be ambiguous since the results of few invasive neuroimaging studies (Mukamel et al., 2010) are not enough to suggest that the functioning of the mirror system in humans is identical to the one in animals.

Non-invasive human studies have shown that the neuronal centers of MNs (premotor cortex, supplementary motor area, parietal cortex, etc.) might be activated while observation of both purposeful actions (Fadiga et al., 1995; Lago and Fernandez-del-Olmo, 2011) and intransitive movement (Borroni et al., 2005; Kemmerer, 2021). Thus, it has been proposed that AONs in the human brain might provide the prediction of the action related feedback (how action must feel and look) and compare the obtained sensory information with the prediction. The last process can be used to correct motor errors that are characterized by mismatch between the prediction and the real sensory feedback (Kilner et al., 2007; Koelewijn et al., 2008; Bonini et al., 2022).

In studies of target-directed movements observation the pre-movement M1 excitability increase was described in Lago and Fernandez-del-Olmo (2011) and Naish et al. (2014) found to be associated with goal recognition. In contrast, observation of intransitive movements cannot involve such pre-movement goal-prediction related changes of excitability. In this case, the effects of intransitive movement observation might be explained only by the sensorimotor resonance activating the specific areas related to involved muscles and corresponding sensory feedback. Thus, human MNs index the observed movement’s kinematics in real time and even predict the future movement trajectory in muscle-specific manner (Gangitano et al., 2001; Lago and Fernandez-del-Olmo, 2011). Alternatively, a recent study (Savaki et al., 2022) suggested that cortical effects of target-directed movement observation might be explained only by kinematics of the movement similarly to the intransitive movements. If true, the modulation of cortical activity by target-directed movement observation might be completely unrelated to target recognition (Hickok, 2013). In this light, it is especially important to differentiate the contribution of different kinematic factors of intransitive movement-being-observed to the activation of AONs.

The biological plausibility of the observing movement was suggested to be one of the main factors affecting AONs activity during action observation in humans. For example (Longo et al., 2008) demonstrated elimination of the automatic imitation effect when participants reacted to the biomechanically impossible actions. Most individuals were capable of successfully recognizing biological motion in the trajectory of animated points of light (Johansson, 1973; Pollick et al., 2002). The results of Shimada and Oki (2012) indicate the enhancement of the MNs activity during observation of an action with slightly unnatural kinematics. Moreover, TMS study performed by Romani et al. (2005) as well as fMRI study from Costantini et al. (2005) demonstrated that premotor and primary motor cortices were activated both observing biologically plausible and biologically implausible movements (i.e., the movements, which are biomechanically impossible). Study (Costantini et al., 2005) further showed modulation of sensorimotor parietal regions while observing biologically implausible movements. In this light, it might seem that there is a differentiation of the functions between different AON regions. Specifically, the role of the parietal regions (within AONs function) might be grounded in the comparison of the movement-being-observed with internal body-map, i.e., mental representation of the corresponding movement (Costantini et al., 2005; Matuz-Budai et al., 2022). Whereas the precentral parts of AONs activate in muscle-specific manner in correspondence with muscles involved in the actual execution of the observed movement regardless of whether they are biologically possible or impossible (Romani et al., 2005).

It is clear, that both the kinematic characteristics and the biological plausibility of observed movements affect the activation of human MNs. However, the results in this area are still controversial: If Costantini et al. (2005) and Romani et al. (2005) have not got any evidence of the participation of the premotor and motor cortices in the detection of biomechanical plausibility of observed movements, then (Calvo-Merino et al., 2006; Heyes and Catmur, 2022) have demonstrated the greater precentral activation when participants viewed movements from their own motor repertoire. Also, they claimed motor cortex inhibition during observation of motor errors and actions with highly unnatural kinematics. Thus, we propose the importance of such basic kinematic attributes of movement as speed, amplitude etc., and the biological plausibility of it in humans MNs activation and motor resonance, but the role of particular cortical parts of AONs in detection of these features of observed movement remains uncovered. This knowledge, in turn, can be beneficial for the action observation therapy usage within motor rehabilitation and motor learning in athletes (Kim et al., 2017; Rungsirisilp and Wongsawat, 2021).

At the same time, we didn’t find any research studying the effects of biological plausibility of observed action on AON-related EEG parameters, such as event-related desynchronization (ERD) which is a sensitive marker of mirror neuron system activity (Lapenta and Boggio, 2014). Overall, ERD in mu and beta frequency bands were shown to be associated with an increase in the activity of somatosensory and motor cortices (Pfurtscheller and Da Silva, 1999). ERD has been reported in studies related to observation of both intransitive and target-directed movements (Muthukumaraswamy et al., 2004; Marshall et al., 2009; Pierno et al., 2009; Streltsova et al., 2010). Articles (Järveläinen et al., 2004; Orgs et al., 2008) reported stronger mu-ERD in the condition when the participant observed previously experienced movements. From a methodological perspective, we highlight that EEG offers good temporal resolution, and this can be successfully used for studying of temporal dynamics of AONs activation in dependence on observed actions speed and amplitude as well as the biomechanical plausibility of them.

We further suggest that a particular difference in observation of biomechanically natural and unnatural movements can appear in visual evoked potentials related to the observed actions. Time-locked EEG activity or event-related potentials (ERP) show the short-termed cortical processing of perceived signals. There are many ERP studies mostly focused on object-oriented actions and goal recognition (Bach et al., 2009; Kutas and Federmeier, 2011), where the amplitude of such ERP components as N400 was modulated by a mismatch between expected and observed tool manipulations. Thus, N400 was proposed as a marker of semantic object-scene inconsistency in observed actions processing (Maffongelli et al., 2020). We hypnotize that N400 could be a marker of incongruity in a broad sense. Thus, while action observation N400 changes can be caused by a mismatch between the sensorimotor mental representation of action and the observed motions. In this way we expect the N400 amplitude increase during the unnatural actions observation and propose the N400 cortical sources could be a part of AONs. Although this ERP’s component is underexplored and wasn’t studied as response to observation of intransitive actions.

Taking everything into account, our study aimed to investigate the effects of anatomical correctness of observed intransitive finger movements on the neuronal activity of sensorimotor cortical areas. First, we attempted to explore the difference in ERD dynamics during observation of anatomically natural and unnatural movements. Second, we tested the differences in N400-like amplitudes between the two conditions. Third, we investigated the dynamics of corticospinal excitability while observing both types of actions. At the same time, we highlight that these parameters might be indexing the activity either of connected parts of AONs or of independent motor-related neuronal processes. In particular, we expected to find a correlation between the ERD of sensorimotor rhythms and N400 amplitude, suggesting that these markers reflect the action congruency processing in AONs. To account for this we suggested explicitly probing whether these measurements are linked to the same neuronal process (or causally linked chain of neuronal events).

The results of this study might be useful for understanding the functioning principles of AON, clarifying the ideas about the mirror system in humans, and for the development of methods for post-stroke rehabilitation using action observation therapy.

## Materials and methods

### Subjects

The study involved 20 right-handed volunteers (11 females, mean age 23 ± 4 years). All volunteers reported no psychiatric and neurological disorders and gave their written consent to participate. All of them were informed about the procedures of the study. The experimental protocol was approved by the Lomonosov Moscow State University Committee for bioethics. The study followed the Declaration of Helsinki Ethical Principles for Medical Research Involving Human Subjects.

### Experimental design

**FIGURE 1:**
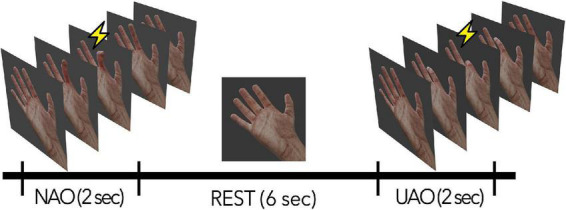
Schematic description of the experimental session. NAO—passive observation of natural actions, UAO—passive observation of unnatural actions. The lightning indicates the phase of movement during which the TMS was applied.

During the experimental session, the subjects were in a comfortable position in a special TMS chair. An 18.5 ‘LCD monitor was placed in front of the subject at 50 cm from their eyes; video stimuli with the image of a virtual anthropomorphic right hand were presented on the screen (see Figure 1). During the session, subjects were instructed to keep their hands completely relaxed and passively observe the video sequences on the screen. The stimuli were video sequences of flexion with the following extension of one of the five fingers of the hand (30 fps, duration 2 s: 1 s for flexion, 1 s for extension). The displayed finger movements could be natural (natural action observation condition, NAO: flexion from 0 to 90 degrees and back) or unnatural (unnatural action observation, UAO: flexion toward the dorsal side from 0 to –90 degrees and back). NAO and UAO stimuli were presented in a semi-random order (2 s - video with movement + 6 s resting state). During the resting state, the subject observed a picture of the motionless hand (see Figure 1).

Within the experimental day, two consecutive sessions were conducted: a session with TMS-evoked MEP registration and a session of an EEG registration. Each of the sessions included a presentation of 30 video stimuli (15 NAO and 15 UAO). The sequence of sessions varied between subjects.

### Electroencephalography

The EEG was recorded using an NVX52 DC amplifier (MKS, Russia) with 30 Ag/AgCl scalp electrodes, placed according to the “10–10” international system in the following positions: Fp1, Fp2, FC5, FC3, FC1, FCz, FC2, FC4, FC6, C5, C3, C1, Cz, C2, C4, C6, CP5, CP3, CP1, CPz, CP2, CP4, CP6, P3, Pz, P4, PO3, PO4, O1, O2. The contact resistance for each of the electrodes was kept below 20 kΩ. The signal was sampled at 500 Hz with a 50 Hz notch filter.

### Transcranial magnetic stimulation

Single transcranial magnetic stimulation (TMS) was performed using a NeuroMS/D stimulator (Neurosoft, Russia). To assess the level of corticospinal excitability, single monophasic magnetic stimuli were applied with a figure-of-eight coil to the area of the left primary motor cortex (M1) at the point with the maximum amplitude of motor responses (“hotspot”) of the targeted muscle—the right hand’s *m.flexor digitorum superficialis* (FDS). Stimulation was performed with a magnitude of 110% relative to the motor response threshold at rest. MEPs from the right hand’s *extensor digitorum communis* (EDC) were also recorded. Coil positioning was controlled by using a neuronavigation system Visor2 (antNeuro, Netherlands). MEPs were registered with EMG electrodes placed on the skin surface of the forearm of the right hand above the FDS as well as the EDC. The ground electrode was installed on the styloid process of the ulna of the left hand. The resistance did not exceed 10 kΩ, the signal was sampled at 2 kHz.

### Analysis

#### Frequency domain analysis of electroencephalography

To extract the components of sensorimotor EEG activity that are sensitive to action observation and to separate them from occipital alpha-rhythm, a spatial filtering procedure using the common spatial pattern (CSP) algorithm was performed (Wang et al., 2006). The method of CSP decomposed the EEG signal into spatial patterns that maximized the difference between the two classes that in our study belong to “active state,” i.e., action observation (both NAO and UAO) and “resting state” (observation of the motionless hand).

To create sensitive filters, the signal of each subject was filtered in individual frequency ranges corresponding to mu (6–15 Hz) and beta (15–30 Hz) frequency ranges, where the ERD was assessed. Then EEG data was divided into 2 s epochs locked to the movement onset: from 0 to 2 s after the movement onset and from 3 to 5 s since the movement termination. First epochs correspond to the “active state,” whereas the second ones were used as resting state. The implementation of the CSP algorithm was taken from the Python library MNE 0.23 (Gramfort et al., 2013) with modifications: as suggested in Cohen (2021), a procedure of cleaning the covariance matrix by removing unrepresentative “noisy” epochs was added.

To extract only EEG features that are corresponding to sensorimotor EEG activity CSP filters with central localization of spatial patterns were selected from the first five columns of the spatial filters matrix, as suggested in Muralidharan et al. (2019). Filters with different, e.g., occipital-parietal localization, were eliminated. The selected filters were applied to the “raw” signal bandpassed in 3–35 Hz. The Morlet wavelet transform was used for the time–frequency analysis. We used a set of complex Morlet wavelets with variable number of cycles for different frequencies. The frequencies of the wavelets ranged from 3 to 30 Hz with 0.3 Hz step, the full-width at half-maximum (FWHM) was equal 140 ms corresponding to a spectral FWHM of 4.5 Hz. The desynchronization value was calculated as the ratio of the signal power in the “action observation” state to the median value of signal power in the resting state; the obtained values were converted to decibels. Negative values corresponded to ERD.

For statistical analysis we used the median values of the signal power within the subject’s individual frequency ranges, where the mu/beta-ERD was observed. We divided the 2 s period of action observation into two equal time intervals: flexion (the first second of AO), and extension (the last second of AO). We further also analyzed the resting state periods both just before the movement, and right after the movement termination. Duration of both resting state intervals included in the analysis were 1 s, thus matching both phases of the AO.

Further, to assess the modulation of the ERD amplitude by action observation phase and by biological plausibility of the observed actions, a two-way repeated-measures ANOVA model with factors Time (df = 3: The last second before AO, the phase of flexion, the phase of extension, and the first second after AO) and Type (df = 1: either natural or unnatural movement) was used. The model included the mean ERD values within all the four levels of factor Time. The Greenhouse-Geisser correction was used to adjust for lack of sphericity. Finally, *post hoc* comparison via two-sided paired *t*-test was performed to compare the differences between NAO and UAO-related ERD in each time interval. The significance level was adjusted using the Bonferroni correction.

#### Event-related potentials analysis

To analyze ERPs, the EEG signal was filtered in the range from 0.1 to 35 Hz. Then, oculomotor artifacts were reduced using fastICA. Specifically, the components highly correlated with Fp1 and Fp2 signals were excluded from the recording. Then, Fp1 and Fp2 channels were dropped. The signal was further re-referenced to common average reference (CAR) and segmented into (–200, 900) ms epochs locked to the movement onset. The interval (–200, 0) ms was used for baseline correction. In order to determine significant differences between ERP under NAO and UAO conditions, we used a non-parametric cluster-level test for spatio-temporal data with 10,000 permutations (Maris and Oostenveld, 2007). Further, the epochs were averaged across the subjects. The difference ERP waveforms were calculated by subtracting the averaged ERP evoked by UAO stimuli from the averaged ERP evoked by NAO stimuli. For signal processing and statistical methods, Python library MNE 0.23 was used (Gramfort et al., 2013).

#### Motor evoked potentials analysis

First, MEPs associated with muscle activity started before the TMS pulse were excluded from the analysis. Also, MEPs deviating from the subject mean amplitude by more than 2 standard deviations were removed from the analysis. On average, 5% of MEPs were removed for each subject. The Friedman test was used to assess the effect of naturalness of observed actions, because the data were non-normally distributed (the Kolmogorov-Smirnov test). Pairwise comparisons with Non-parametric Wilcoxon sign-rank tests were performed to compare the differences between conditions. Statistical tests were conducted in the SciPy module (ver. 1.4.1) in Python (ver. 3.7).

We also used Pearson’s correlation test to find the relations between MEP amplitudes, ERD values and amplitudes of ERP components.

## Results

### Event-related desynchronization dynamics

**FIGURE 2:**
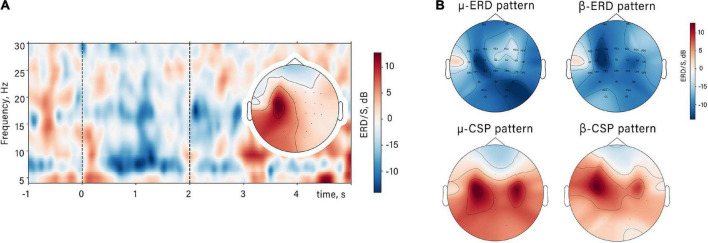
The spatio-temporal mu/beta-ERD patterns. **(A)** Time-frequency dynamic of spectral power in contralateral mu-ERD spatial source in a single subject in NAO condition and its spatial topography. Dashed lines restrict the time interval of action observation. **(B)** Averaged ERD/S value distribution and averaged sources of the mu/beta-ERD (CSP patterns) for all subjects.

We observed mu and beta desynchronization during the action observation unlike the resting state condition with observation of motionless opened palms. ERD appeared with the start of both natural and unnatural flexion (Figure 2). Two subranges within mu and beta activity were identified: averaged by subjects’ range of mu-ERD—8–14 Hz, beta-ERD—17–25 Hz. For these subranges, spatial filters were obtained. ERD reaction had typical parietal-central localization (bilaterally with dominance in contralateral side relative to the observed hand). Such spatial distribution of ERD can be observed both on the averaged topographic map obtained for the raw signal before CSP and on the averaged sources of the selected CSP patterns (Figure 2).

**FIGURE 3:**
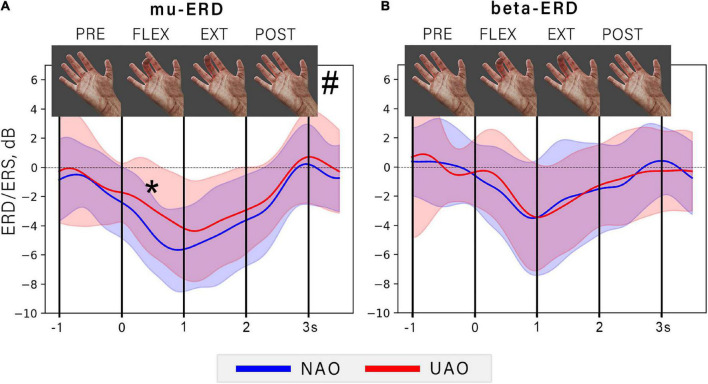
The dynamic of averaged ERD during NAO (blue line) and UAO (red line) conditions. The graph shows the EEG power time course for mu **(A)** and beta **(B)** frequency bands. Color shapes show corresponded mean ± std range. The vertical lines limit the 1-s-long consequent time intervals: PRE, rest period before action observation; FLEX, flexion (the first second of AO); EXT, extension (the last second of AO); POST, rest period after AO. At the top of the figure the particular frames of demonstrated movement are shown. Asterisk indicates a significant difference (*p* = 0.006) between mu-ERD amplitudes in NAO and UAO conditions during observation the flexion phase of demonstrated action, grid symbol indicates a significant effect of the type of observed action on the ERD amplitude (*p* = 0.0023).

It can be noticed that ERD amplitude changes during action observation synchronously with amplitude of the observed flexion. Specifically, ERD increases during the first movement phase and reaches a peak simultaneously with the maximum finger flexion angle. During observation of extension ERD decreases and reaches the baseline at the moment of action termination (see Figure 3).

Two-way repeated-measures ANOVA revealed a significant effect of the phase of the observed movement on the ERD amplitude in mu (*F* = 32.24, *p* < 0.00001, $\eta_{p}^{2}$ = 0.62), and in the beta frequency range (*F* = 18.24, *p* < 0, 00001, $\eta_{p}^{2}$ = 0.52). However, the type of observed action (either natural or unnatural) significantly affected only mu-ERD (*F* = 12.32, *p* = 0.0023, $\eta_{p}^{2}$ = 0.39). Specifically, the NAO condition was characterized by a greater mu-ERD than the UAO: the *post-hoc* tests revealed a significant difference during observation of finger flexion, i.e., on the first action phase (T-statistics = –3.1, *p* = 0.006).

### Motor evoked potentials amplitude

**FIGURE 4:**
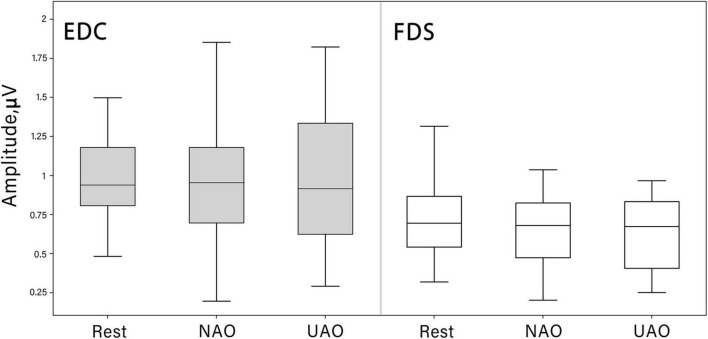
Changes in motor evoked potential (MEP) peak-to-peak amplitude (mV) during AO in different conditions (median shown as the middle horizontal line, the upper and lower quartiles displayed as boxes, and IQR interval is represented as whiskers). TMS was always given to the left motor cortex contralateral to the observed hand. MEPs were recorded from FDS (white boxes) and EDC (gray boxes) muscles.

MEP analysis did not reveal an increase in MEP amplitude in both conditions of AO as compared with the resting state: no significant differences emerged among the three conditions (Rest, UAO, NAO) at the Friedman test for FDS [χ2 (2) = 1.36, *p* = 0.5] as well as EDC [χ^2^(2) = 0.31, *p* = 0.85] (see Figure 4).

### Event-related potentials

**FIGURE 5:**
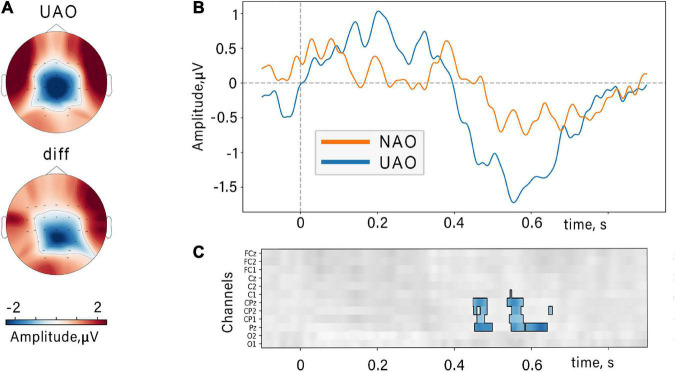
Event-related potentials locked to the movement onset. **(A)** Topographic grand average map of UAO-related N400 peak and topographic difference (“diff”) of the N400 peak (UAO minus NAO). **(B)** N400 components related to NAO (orange) and UAO (blue) conditions (averaged over all subjects and channels included in the revealed cluster). **(C)** Results of cluster-based permutation tests: Significant difference between NAO ERPs and UAO ERPs for group of subjects at midline and nearest electrodes and time slice. Color coding is shown for difference ERP waves (UAO-NAO), insignificant differences (*p* > 0.05) are shown in gray.

A non-parametric cluster-based permutation analysis indicated an effect of biological plausibility on ERP amplitude. The revealed cluster corresponded to slow late negative potential within 400–700 ms range (see Figure 5). This component is characterized by a larger amplitude during UAO. The cluster included EEG channels from the midline of the central parietal region (CP1, CPz, CP2, Pz).

We also analyzed the correlations between the amplitude of the MEP amplitude, mu/beta ERD amplitude, and the amplitude of the late ERP component. Importantly, no significant associations between these parameters were found.

## Discussion

In the current study, we examined oscillatory and phase-locked EEG activity, as well as corticospinal excitability, of healthy subjects during observation of intransitive actions. We assessed the effects of anatomical possibility of observed movements on these markers of AONs activity by demonstrating natural flexions of fingers (NAO) as well as unnatural flexions (UAO) when fingers wriggled out violating the biomechanical constraints of palm anatomy.

The main result of the present study is the weaker mu-rhythm desynchronization during observation of unnatural actions compared to natural. Conversely, the N400 amplitude was significantly larger in UAO condition.

### Dynamics of mu- and beta-event-related desynchronization

Our results showed that mu and beta ERD develops during simple fingers flexion observation. It is consistent with the findings of several brain imaging studies demonstrated sensorimotor and premotor cortical areas activation not only during observation of goal-directed actions but during intransitive action observation as well (Babiloni et al., 2002; Pfurtscheller et al., 2007; Lesourd et al., 2018). Moreover, Aleksandrov and Tugin (2012), showed no significant differences between sensorimotor activation during observation of movements performed with or without a goal and even movements using a tool. We observe a temporal evolution of ERD value (in both mu and beta bands). The temporal dynamic of ERD developed synchronously with flexion angle changes: ERD value reached the maximum at the moment of the flexion end, when the movement amplitude was the largest. Importantly, occipital alpha modulated by visual attention cannot be a reason for such ERD dynamic because for analysis we selected only CSP filtered oscillatory activity from central-localized sources, whereas filters with occipital coordinates were removed. Therefore, the observed dynamics likely corresponds to sensorimotor activity exactly. Importantly, the sensorimotor EEG rhythms reflect activation of precentral and postcentral cortical regions (Szurhaj et al., 2003). Accordingly, we suggest that an increase of activity in these regions while action observation might arise due to the activity of mirror neuron networks. Thus, our results confirm that the human MNs are sensitive to simple aimless actions, and their activity is modulated by the observed movement profile.

We suggest that the revealed ERD pattern and its relation to the movement dynamics might reflect the processing of observed actions at the level of motor patterns in the sensorimotor cortex, analogously to the real movement performance (Avanzini et al., 2012). Importantly, the dependency of mu/beta ERD on the kinematic parameters of executed and imagined movement has been previously observed (Cassim et al., 2000; Gu et al., 2009). Moreover, authors of Avanzini et al. (2012) and Luo et al. (2018) revealed a similar relation between ERD dynamics and observed action. Although, these studies suggested modulation of ERD value only by observed movement velocity, whereas we extended their findings to other parameters of the kinematic domain, showing the dependence of the ERD value on flexion amplitude. Specifically, ERD increased during the flexion, and decreased during extension returning to baseline at the very end of the observed movement; whereas both flexion and extension of observed fingers had the same velocity. Taking everything into account, our result complements the previous studies (Avanzini et al., 2012) suggesting that the amplitude of mu and beta-ERD also reflects the amplitude and the limb pose, i.e., contraction strength of the observed limb.

Such dynamical suppression of the mu and beta rhythms during action observation, could be due to a neurophysiological mechanism of sensorimotor resonance. Considering these findings, we might suggest that the response is likely to be less prominent in case of observation of anatomically unnatural actions due to the less sensorimotor resonance (Coll et al., 2015; Riečanský et al., 2020). Indeed, the comparison of NAO and UAO revealed that the mu-ERD reaches a greater value during NAO. This effect was significantly manifested while observation of the flexion stage. At the same time, no differences between NAO and UAO conditions in beta-ERD amplitude were observed (see Figure 3). In light of this, we suggest that it is possible that sensorimotor oscillations within beta and mu bands can reflect different characteristics of observed actions. As described in action observation and motor imagery studies, the amplitude of alpha and beta oscillations often changes synchronously (Babiloni et al., 2002) but there is evidence of functional dissociation of these rhythms (Crone et al., 1998; Shibuya et al., 2021): The mu-ERD rather reflects the activity of the somatosensory cortex, and the beta-ERD stands for the activity of the precentral cortex regions (Crone et al., 1998). It is in line with defined topography of areas where mu and beta-ERD were found, and with topography of corresponding forward CSP patterns: the largest mu-ERD was detected over centro-parietal sites (Ñ-CP channels), whereas beta-ERD was localized more frontally, predominantly over FC-C sites (see Figure 2).

It has been proven that during action observation, mu-ERD is modulated by the mental image of the sensory components of the observed movement and is determined by the sensory experience of the subject (Coll et al., 2015, 2017); thus, the lower mu-ERD in the UAO condition can indeed be explained by the fact that the anatomically unnatural movements cause less somatosensory resonance in the mirror networks. Thus, we speculate, less resonance during UAO can be related with subjects’ somatosensory experience. The research of effects of motor and sensory experience reported greater mu/beta desynchronization during observation of familiar, experienced action (Quandt et al., 2012, 2013; Quandt and Marshall, 2014). However, the role of sensorimotor experience in mu/beta ERD caused by action observation remains unclear (Babiloni et al., 2010). In particular studies (Calvo-Merino et al., 2005, 2006) found greater MNs activity when subjects viewed moves from their own motor repertoire, thus, we could expect that NAO will be characterized by stronger mu-ERD as well as stronger beta-ERD compared with UAO. On the contrary, our results indicate the same dynamic in beta-ERD in both conditions. We propose the different role of somatosensory and precentral cortical areas in action recognition: motor and premotor cortices resonate in relation to muscles involved in execution of the observed movement (that is in line with (Romani et al., 2005), whereas activation of parietal somatosensory regions depended on sensory familiarity of action and can discriminates impossible from possible movements (Costantini et al., 2005). Thus, the motor cortex activation is rather related to coding the specificity of the body part then to action kinematic plausibility (Romani et al., 2005). In turn, the relevant information about the movement plausibility derives from the parietal cortex (Romani et al., 2005). The centro-parietally distributed N400 discovered in present work supports this hypothesis.

Finally, it is important to note, the temporal dynamics of mu/beta-ERD was similar in both NAO and UAO. Thus, the sensorimotor areas resonated even during the observation of unrealistic movements that were never present in the human experience in a similar way as during natural familiar actions.

### Changes in the event-related potentials amplitude

Along with prominent changes in the ERD, we observed that action stimuli elicited late negative potential with peak latency at 400–700 ms relative to the movement onset (see Figure 3). This component was significantly larger if subjects observed anatomically unnatural actions.

However, we note that the disambiguation time between NAO and UAO stimuli is not accurately controlled, and we propose a large jitter of the onset latency between trials. In other words, despite the fact, that ERPs are locked to the movement onsets, the beginning of the ERPs are likely to correspond to a short time interval, during which NAO- and UAO-related cannot be distinguished. Since both types of movements are initiated gradually from the same starting point (rest position), the disambiguation time is likely dependent on the subject and their visual attention. Taking it into account, no precise latency-based classification of ERP components is available. However, we suggest that the significantly different ERP component is likely to represent *N400-like activity*. Though the N400 component was traditionally described as a marker of semantic dissociation during language comprehension (e.g., Kutas and Hillyard, 1980; Berkum et al., 1999), the recent studies showed the N400 effect in a broader range of situations. For instance, Sitnikova et al. (2008) and Bach et al. (2009) showed enhanced N400 during observation of motor acts belonging to everyday activities with incongruent objects (e.g., shaving with a rolling pin). Moreover, Sitnikova et al. (2008) defined negative potential in a similar time window (350–600 ms) as N400-like activity. Taking it into account, the stimuli used in our study are of a particular interest since it is not the object-being-perceived, which is incongruent with regard to a particular movement, but the movement *per se* in being incongruent with no respect to any object. Thus, our results support more general conceptualization of the N400 component. The fact that the N400-like activity is present in all the discussed cases seem to contradict the N400 theories, which imply more “narrow-band” N400 genesis (Duncan et al., 2009). On the other hand, our results are fully in accordance with suggestions that N400 represent interaction (or rather a particular conflict) between bottom-up stimulus-perception activity with top-down neuronal network shaped by the participant’s expectation based on their long-term experience (see Federmeier and Laszlo, 2009; Kutas and Federmeier, 2011). Accordingly, it is likely that N400-related semantic dissociation should be further interpreted in a broader supralinguistic sense.

We found centro-parietal midline localization of the peak maximum of discovered N400-like potential. That is consistent with data on the localization of N400 sources (Khateb et al., 2010; Balconi and Vitaloni, 2014). It has been suggested that the parietal areas may store spatial and kinesthetic information about movements and map these representations onto the premotor and motor cortex, that in turn contain the concrete motor programs (Sirigu et al., 1996). Moreover, (Costantini et al., 2005) found a selective increase of the BOLD signal in the posterior parietal cortex (PPC) during observation of impossible human actions (very similar to used here in UAO condition), and they propose such activation as the reaction on mismatch between somatic mapping of visual input and covert action imitation. During action observation, the parietal cortex plays an integrative role: it receives visual information from the occipital cortex, somatosensory information from S1, and motor information from motor cortex areas (Wise et al., 1997). Our data contribute to the idea proposed in Babiloni et al. (2002), that such integrative processes would be important for matching the observed action among sensorimotor and postural memories. We suggest that such an integrative role of PPC within action recognition can be discovered by studies using movies with simple aimless movements.

### Absence of motor evoked potentials increase

Previous studies demonstrated a MEP amplitude increase during observation as target-directed actions (e.g., grasping or pointing) (Gangitano et al., 2001; Spaccasassi et al., 2022) as well as intransitive movements (Borroni et al., 2005; Lagravinese et al., 2017). However, in our study, where we used stimuli with simple intransitive movements, we didn’t find any difference in MEPs amplitude between rest condition and both AO conditions.

The AO effect might have not been observed due to the limitation of the current study. Specifically, according to Borroni et al. (2005), cortical excitability during action observation changes dynamically and depends on the phase of the observed movement. Then, no effect shown in the present study may be associated with the choice of the phase of application of the TMS pulse (at the moment of the end of flexion before extension, at 900 ms after the movement onset). We choose such TMS applying time based on (Moriuchi et al., 2017), when flexion amplitude is maximal. Although, at this phase demonstrated action stopped before the finger extension starts. Such a methodological approach might lead to masking the expected excitability increase. It may be suggested that the increase of cortical excitability in particular in areas corresponding to FDS develops during observation of flexion and disappears toward the end of this action’s part. According to Lapenta et al. (2018) the M1 activation corresponds to maximum hand aperture velocity, but not to the end of object lifting, when muscle’s contraction is largest, but static. Significant excitability increase during mid-phases of the action but not during maximal muscles contraction indicate that M1 activation anticipates movement with a time interval which reflects the temporal resolution of ability of AONs to predict action outcomes (Borroni et al., 2005; Lapenta et al., 2018).

For the future studies, we recommend using several time-points for TMS applying with regard to the observed movement phase to avoid the methodological omission made in current research.

### Correlation between event-related desynchronization, motor evoked potential, and event-related potentials amplitudes during action observation

We found no **correlation** between mu/beta ERD values and MEP amplitudes. This finding complements the work by Lapenta et al. (2018), who showed no correlation between these parameters while observing reach-to-grasp actions. We propose several lines of interpretation to account for no correlation revealed between the metrics both associated with sensorimotor cortical activity. First, we note that mu-ERD is an index on somatosensory activity and its value may have non-linear relations with such M1 excitability markers as MEP amplitudes. Specifically, TMS explores the activity of the primary motor cortex, which may not always be activated by MNs, and may not be involved in the analysis of simple actions unlike visual and somatosensory cortices. Alternatively, it is possible that the peak of desynchronization and an increase in excitability occur at different phases of the movement (Gangitano et al., 2001; Grosprêtre et al., 2016; Spaccasassi et al., 2022). Importantly, it was shown that ERD evolution is also phase-dependent and sensorimotor excitability dynamics can be reflected in MEPs amplitude and ERD value with different latency (Lapenta et al., 2018). At the same time, it is important to emphasize that we analyzed the correlation between ERD values and MEPs obtained on an inter-subject level by averaging the values within each individual. Accordingly, such an approach cannot reveal trial-by-trial fluctuations of desynchronization and corticospinal excitability if present. We find it to be the limitation of our study and suggest future studies to consider using TMS-EEG co-registration. Although, TMS-EEG co-registration study by Spaccasassi et al. (2022) found no intra-subject correlation between these indices. Following, they suggested that ERD and MEPs reflect distinct neural mechanisms of motor resonance (Spaccasassi et al., 2022).

Both mu-amplitude and N400 amplitude have demonstrated the sensitivity to observed action plausibility, and we expected to find any correlations between these markers. Though, no correlations were found between N400 amplitudes and the amplitude of mu desynchronization. The N400 amplitude maximum was found over the CPz channel, and the greater difference between conditions appeared in the Pz channel. Such scalp distribution of the N400 ERP-component points to the involvement of the parietal cortex in the N400 generation, which is in line with other source localization studies (Balconi and Vitaloni, 2014). At the same time, the ERD values peaking around central brain areas correspond to somatosensory and motor cortices. Alternatively, the neurons of the parietal cortex producing altered N400 response for the UAO might be a part of the action observation network (Rizzolatti and Sinigaglia, 2010); however, their activity may not be directly related to the activity of the sensorimotor areas. For example, N400 sources can be a part of a special system that matches observed actions with the internal body-map (Amoruso et al., 2013).

## Conclusion

We discovered that passive observation of simple actions led to desynchronization of EEG sensorimotor rhythms, which developed synchronously with the flexion amplitude. Moreover, mu-ERD and centro-parietal N400 were sensitive to anatomical incongruence of observed movements, while beta-ERD amplitude was not affected by the naturalness of action. Thus, our data point to functional specificity of different cortical nodes within AONs underlying the mirroring of the different features of the observed action. These results might be also used to broaden the conceptual framework of the N400 such that to account not only for the observation of semantic inconsistencies of a particular action, but also for its overall conformity with the participant’s sensorimotor experience. Accordingly, N400 can be an important marker of AONs activity during action recognition in the further studies.

## Data availability statement

The raw data supporting the conclusions of this article will be made available by the authors, without undue reservation.

## Ethics statement

The studies involving human participants were reviewed and approved by the Lomonosov Moscow State University Committee for bioethics. The patients/participants provided their written informed consent to participate in this study.

## Author contributions

NS and DB conceived of the presented idea. NS developed the theory and processed the data. NS, LY, and AM carried out the experiment. AK supervised the project. All authors discussed the results and contributed to the final manuscript.
